# Sulfobetaine methacrylate hydrogel-coated anti-fouling surfaces for implantable biomedical devices

**DOI:** 10.1186/s40824-017-0113-7

**Published:** 2018-02-12

**Authors:** Se Yeong Lee, Yunki Lee, Phuong Le Thi, Dong Hwan Oh, Ki Dong Park

**Affiliations:** Department of Molecular Science and Technology, Ajou University, San 5, Woncheon, Yeongtong, Suwon, 16499 Republic of Korea

**Keywords:** Surface modification, Zwitterions, Anti-fouling properties, Fenton reaction, Hydrogel

## Abstract

**Background:**

Zwitterionic molecules have been widely studied as coating materials for preparing anti-fouling surfaces because they possess strong hydration properties that can resist non-specific protein adsorption. Numerous studies on surface modification using zwitterionic molecules have been investigated, such as electrochemically mediated and photoinitiated radical polymerization. However, these methods have some limitations, including multi-step process, difficulties in producing thick and dense layers as well as the requirement of extra facilities. In this study, we report a novel zwitterionic hydrogel-coating method via Fenton reaction for the preparation of anti-fouling surfaces.

**Methods:**

Sulfobetaine methacrylate (SBMA) hydrogel was coated on polyurethane (PU) by polymerization of SBMA molecules via the Fenton reaction. The coated surfaces were characterized by the measurements of water contact angle, SEM and XPS. The anti-fouling properties of the modified surfaces were evaluated by reductions of fibrinogen absorption and cell (human dermal fibroblasts, hDFBs) adhesion.

**Results:**

SBMA hydrogel layers were coated on the PU substrates and these layers have a high affinity for water. The hydrogel coatings were highly stable for 7 days, without a significant change in surface wettability. Importantly, the hydrogel-coated PU substrates decrease 80% of surface-adsorbed fibrinogen and surface-attached hDFBs (compared with uncoated PU substrates), indicating the excellent anti-fouling activities of modified surfaces.

**Conclusions:**

The hydrogel-coated PU surfaces prepared by Fenton reaction with anti-fouling properties could have potential uses for implantable biomedical devices.

## Background

Biomaterials are inert natural or synthetic materials that come in contact with tissue, blood or biological fluids, and are intended for use in prosthetic, diagnostic, therapeutic or storage applications without adversely affecting the living organism and its components [1]. There are various types of biomaterials, such as metals, ceramics and polymers. The biomaterials have been widely used for biomedical applications, such as blood-contacting devices, biosensors, drug delivery vehicles and other implantable devices. When being implanted into the body, various reactions can be happened between the host body and biomaterials. For examples, the non-specific protein adsorption onto the implant surfaces, subsequently aiding the blood cell adhesion, which leads to the thrombus formation and causes detrimental clinical complications [2]. In this context, surface modification with protein/cell-resistant properties is an effective strategy to improve the in vivo performance of biomaterials used for blood-contacting devices [3–5]. Through a proper modification technique, the biomaterial surfaces can be physically or chemically modified to minimize protein adsorption and cell adhesion.

Hydrophilic polymers such as poly(2-hydroxyethyl methacrylate) and poly(ethylene glycol) (PEG) are routinely used to modify surfaces. After being immobilized on the surfaces, these materials create a hydration layer on the surfaces, thereby improving the protein-resistant properties of the modified surfaces. Although the anti-fouling properties of PEGylated surfaces were greatly enhanced, several disadvantages have been reported for in vivo application, such as the relatively poor performance in blood serum/plasma and susceptibility to oxidation [6, 7]. Zwitterionic polymers, a family of materials possessing strong hydration properties that effectively resist non-specific protein adsorption, have been extensively studied for preparing anti-fouling surfaces [8–10]. Compared to PEG, zwitterionic polymers are not only biomimetic but also biocompatible and non-cytotoxic, as their endotoxin levels were found to be acceptable for in vivo implantation [11]. Numerous approaches are available to modify a surface using zwitterionic molecules, such as photoinitiated cross-linking and electrochemically mediated radical polymerization. However, these methods have some limitations, including multi-step process, use of toxic reagents, difficulties in producing thick and very dense layers, in addition to the requirement of extra facilities [12–15].

Recently, free radicals generated by redox systems under mild conditions have been applied to initiate the crosslinking process. Among them, the redox pair of ferrous salt and hydrogen peroxide (Fenton reaction) produces highly reactive hydroxyl radicals, as shown in the equation below [16]:

$$ {\mathrm{Fe}}^{2+}+{\mathrm{H}}_2{\mathrm{O}}_2\to {\mathrm{Fe}}^{3+}+{\mathrm{O}\mathrm{H}}^{-}+{\mathrm{O}\mathrm{H}}^{\bullet } $$

Nowadays, the Fenton reaction is used to handle water pollution [17]. Also, Fenton’s reagent has been used to induce radical polymerization of vinylic molecules for more than half century [18]. Fenton reaction was recently explored to prepare poly (N-vinyl-2-pyrrolidone) (PVP) hydrogels, which did not show any toxic or disturbing outcomes based on a dermal inflammation test in rabbits [19].

Herein, we report a simple and effective method to develop anti-fouling surfaces, by coating a zwitterionic hydrogel onto polyurethane (PU) substrates. We hypothesized that the high hydration of both the zwitterionic polymer and hydrogel network would suppress the protein absorption and thus prevent the unwanted reactions between cells and surfaces. The hydrogel was rapidly formed by Fenton reaction-initiated free radical polymerization of sulfobetaine methacrylate (SBMA) and ethylene glycol dimethacrylate (EGDMA). The physicochemical properties of the modified surface such as surface morphology, chemical composition, and surface wettability were characterized by scanning electron microscopy (SEM), X-ray photoelectron spectroscopy (XPS), and water contact angle measurement, respectively. The anti-fouling activities of hydrogel coated surfaces were investigated by in vitro protein adsorption and cell attachment.

## Methods

### Materials

PU (Estane, 60D, Lubrizol Corporation, Wickliffe, USA) was kindly provided by Genoss, Suwon, Korea. SBMA, iron (II) chloride (FeCl_2_), L-ascorbic acid (AA), cumene hydroperoxide (CHP), ethylene glycol dimethacrylate (EGDMA), hexane, sodium dodecyl sulfate (SDS), and tetrahydrofuran (THF) were obtained from Sigma-Aldrich (St. Louis, MO, USA). N, N-dimethylacetamide (DMAc) was supplied by Junsei Chemical Co. (Tokyo, Japan). All other chemicals were purchased from Sigma–Aldrich unless otherwise specified.

For the protein adsorption measurement, horseradish peroxidase (HRP)–conjugated anti-IgG, fibrinogen, anti-fibrinogen, 3,3′,5,5′-tetramethylbenzidine (TMB), bovine serum albumin (BSA) and sulfuric acid were purchased from Sigma–Aldrich. For the cell study, Dulbecco’s modified Eagle’s medium (DMEM), fetal bovine serum (FBS), penicillin-streptomycin (P/S), trypsin/ethylenediaminetetraacetic acid (EDTA) and Dulbecco’s phosphate buffered saline were purchased from Gibco BRL (Grand Island, NY, USA). The DNA-specific fluorochromes, 4′-6-diamidino-2-phenylindole (DAPI) was procured from Vector Laboratory (Burlingame, CA, USA).

### Preparation of PU substrates

PU substrates were prepared using casting and solvent evaporation. PU pellets were dissolved in a co-solvent of THF:DMAc (50:50, *v*/v) at 60 °C. The PU solution (10% *w*/*v*) was poured into a glass mold at 60 °C and dried under vacuum for 48 h. Before modification, PU substrates were cut out with a metal stamp and washed with deionized water (DW, 48 h), to extract residual solvents. The thickness of PU substrates was measured using a digital caliper (Mitutoyo Co., Japan).

### Preparation of SBMA hydrogel-coating on PU substrates

**Fig. 1:**
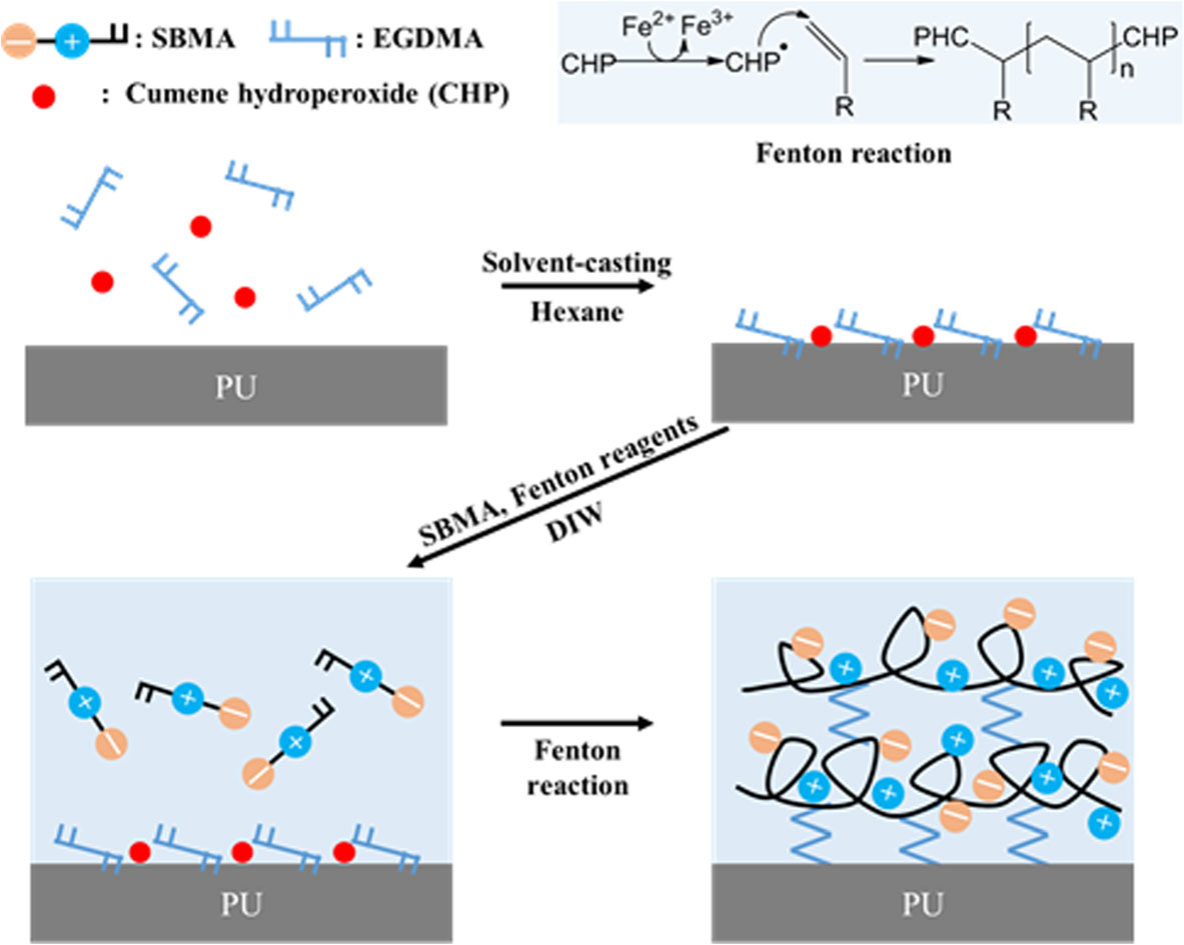
Schematic presentation for preparation of SBMA hydrogel-coated PU substrates

Hydrogel-coated PU substrates were fabricated by a two-step process (Fig. 1). First, the PU substrates were immersed in a hexane solution containing EGDMA (5%, *v*/v) and CHP (20%, v/v) for 5 min at room temperature (RT). Then, the samples were placed into a solution containing SBMA (50%, *w*/*v*), FeCl_2_ (0.1%, *w*/*v*), and AA (1%. w/v) for 15 min at RT. After the coating procedure, the hydrogel-coated PU substrates were washed with 1% (w/v) SDS solution for 5 min to remove unbound SBMA and immersed in DW overnight.

### Surface characterization of SBMA hydrogel coated PU substrates

The morphology of the hydrogel-coated PU substrates was examined by scanning electron microscopy (SEM, S-800, Hitachi). Briefly, the substrates were affixed onto aluminum stubs using double-sided adhesive conductive carbon tape. Before imaging, the samples were coated with a very thin layer of gold. The images were captured under high vacuum conditions at 5 kV.

The hydrophilicity of the modified surface was measured by the sessile drop method, using a contact angle goniometer (GBX Inc., France). A 1 μL water drop was placed on the surface using a micro-syringe. The water contact angles were measured at different positions on the surface after 30 s of dropping.

The surface composition of the hydrogel-coated PU substrates was analyzed by X-ray photoelectron spectroscopy (XPS) (Thermo Electron, K-Alpha, USA). The C_1s_ hydrocarbon peak at 284.84 eV was used as the reference for all binding energies. Measured peak areas were converted to normalized peak intensities by atomic sensitivity factors, from which the atomic compositions of surfaces were calculated.

### Stability of hydrogel coating

The stability of the hydrogel coating on PU surfaces was investigated by measurement of the water contact angle using the contact angle goniometer as described above. Before the analysis, the coated surfaces were immersed in distilled water for predetermined time periods (0, 1, 3, 5, 7, 14 and 21 days), and then air-dried overnight at RT.

### In vitro anti-fouling evaluation

#### Fibrinogen adsorption

The fibrinogen adsorption onto the bare and SBMA hydrogel coated PU surfaces was evaluated using the the antigen-antibody reaction method. Fibrinogen was dissolved in PBS at 1 mg/mL. The surfaces were first immersed in PBS solution for 30 min to achieve equilibrium and then transferred to a 24-well plate which contained 500 μL of fibrinogen solution per well. The experiment was preceded at 37 °C for 3 h. Next, the samples were washed (×3) with PBS solution containing 0.05 wt.% TWEEN 20 (PBST) for 5 min. In order to prevent the noise signal from non-specific fouling, the samples were blocked with 2 mg/mL BSA in PBST for 30 min. Afterward, the samples were separately immersed in solutions containing anti-fibrinogen at 1:10,000 dilution for 1 h at RT. After removal from the primary antibody solutions, the samples were washed (×3) with PBST for 5 min, followed by incubation in corresponding HRP-conjugated IgG at 1:10,000 dilution for 1 h at RT. After washing (×3) with PBST for 5 min, the samples were reacted with TMB substrate for 10 min and the reaction was stopped with H_2_SO_4_ (1 M, TMB:H_2_SO_4_ = 2:1 *v*/v). The optical density (OD) of the supernatants was read at 450 nm by a microplate reader (SpectraMax, Molecular Devices, Sunnyvale, CA) to determine the amount of adsorbed fibrinogen.

#### Cell adhesion

To evaluate cell adhesion onto the bare and hydrogel-coated PU substrates, human dermal fibroblasts (hDFBs) were seeded on the PU surfaces at a density of 2 × 10^4^ cells/cm^2^. The cell-seeded PU substrates were cultured with DMEM supplemented with 10% FBS containing 1% P/S, under standard cell culture conditions (5% CO_2_, at 37 °C). After 6 h of incubation, blue nucleic acid staining was performed with DAPI which preferentially bind to A (adenine) and T (thymine) regions of DNA. The DAPI-stained cells were imaged by fluorescence microscopy (TE-2000, Nikon, Japan).

Automatic cell counting in the fluorescence images was performed using the ImageJ software (NIH, Bethesda, MD, USA). Five images per samples were used to analyze the cell number by counting the nuclei of cells. The entire area of the image was calculated and the result was presented as the number of cells per unit area of a sample.

### Statistical analyses

Experimental data were analyzed by the Student’s t-test. Statistical significance was set at having ^*^P < 0.05. All the experiments were performed in triplicate, and data were presented as the mean ± SD.

## Results and discussion

### Surface characterization of SBMA hydrogel-coated PU substrates

**Fig. 2:**
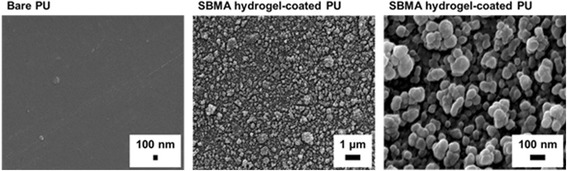
Surface morphology of PU and SBMA hydrogel-coated PU substrates

The morphology of bare and hydrogel-coated PU surfaces was visualized by SEM. As shown in Fig. 2, the surface roughness increased significantly after coating hydrogel onto the PU surfaces. Furthermore, the hydrogel coating was composed of small SBMA spheres, which adhered to each other. This result was explained by the effect of EGDMA cross-linkers, which could interact with two SBMA chains and lead to the formation of a sphere-like network [20].

**Fig. 3:**
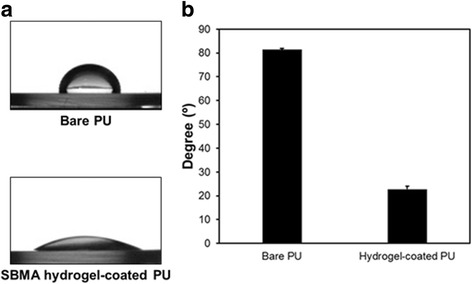
Surface wettability (**a**) and water contact angles (**b**) of bare and SBMA hydrogel-coated PU substrates

The hydrophilicity of the PU surfaces was investigated before and after coating with the SBMA hydrogel, using the static water contact angle measurement. Figure 3 shows the dramatic decrease in the water contact angle of the SBMA hydrogel-coated PU surfaces (81.5° ± 0.6°) compared to the bare PU ones (22.7° ± 1.4°). This result indicated the successful coating of hydrophilic zwitterionic hydrogel onto the PU substrates.

**Fig. 4:**
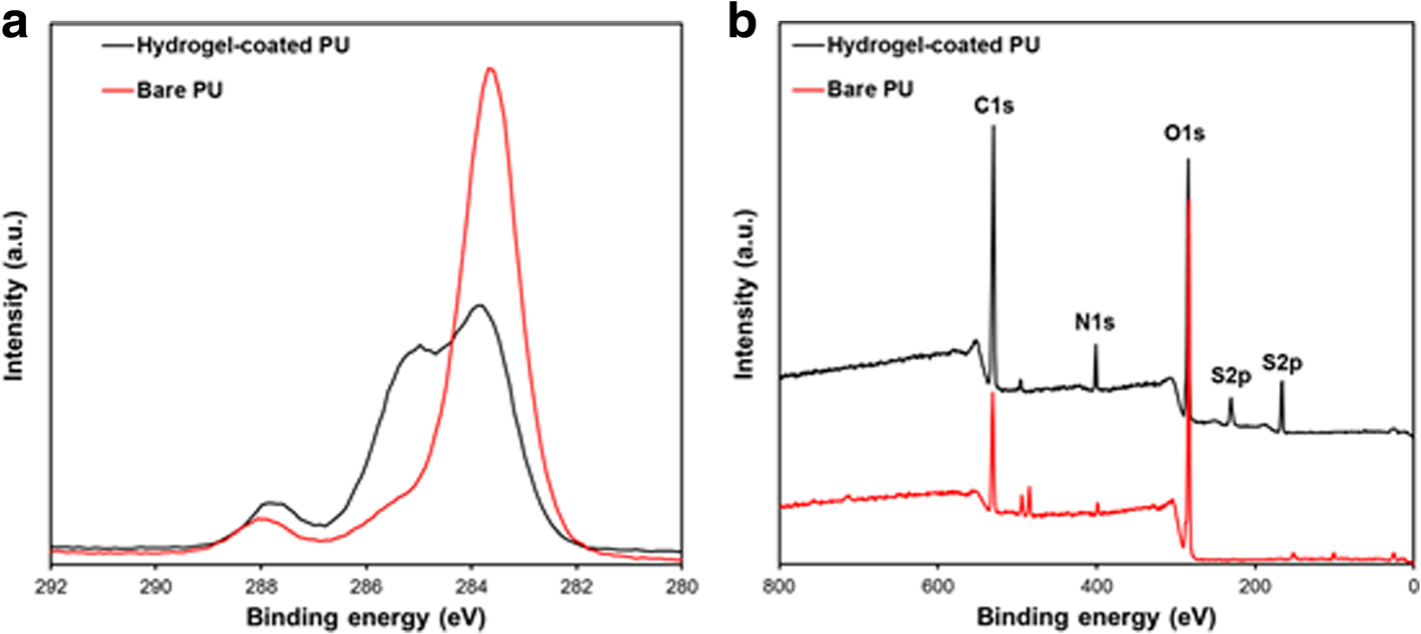
XPS C_1s_ core-level spectra (**a**) and XPS wide-scan spectra (**b**) of bare and SBMA hydrogel-coated PU surfaces

The difference in chemical composition between the bare and hydrogel-coated surfaces was determined by XPS analysis. Figure 4a shows the XPS C_1s_ core-level spectra processed via curve fitting. There was a high binding energy peak at 288 eV, corresponding to the O-C=O species. The main C_1s_ peak for SBMA is attributed to the overlap of CO, CN^+^, CC, and CSO_3_^−^ (~285.2 eV) [21]. In the wide-scan XPS spectra (Fig. 4b), after the polymerization of SBMA onto the surface of PU substrates, the new peaks appeared at around 401.1 eV (N_1s_) and 167.1 eV (S_2p_), which originated from the -(CH_3_)_3_N^+^ and -SO_3_^−^ groups of SBMA molecules. These data demonstrated that SBMA hydrogel was successfully coated onto the PU surface. Both the nitrogen and sulfur compositions calculated from XPS spectra concur with the stoichiometric compositions (Table 1).

**Table 1:** Surface chemical composition of bare and SBMA hydrogel-coated PU substrates

Sample	Atomic percentage (%)				Atomic ratio
	C1s	O1s	N1 s	S2p	N/S ratio
Bare PU	85.64	13.25	1.11	0	–
Hydrogel-coated PU	64.16	25.63	4.71	5.5	0.86

SBMA, sulfobetaine methacrylate; PU, polyurethane

### Stability of hydrogel coating

**Fig. 5:**
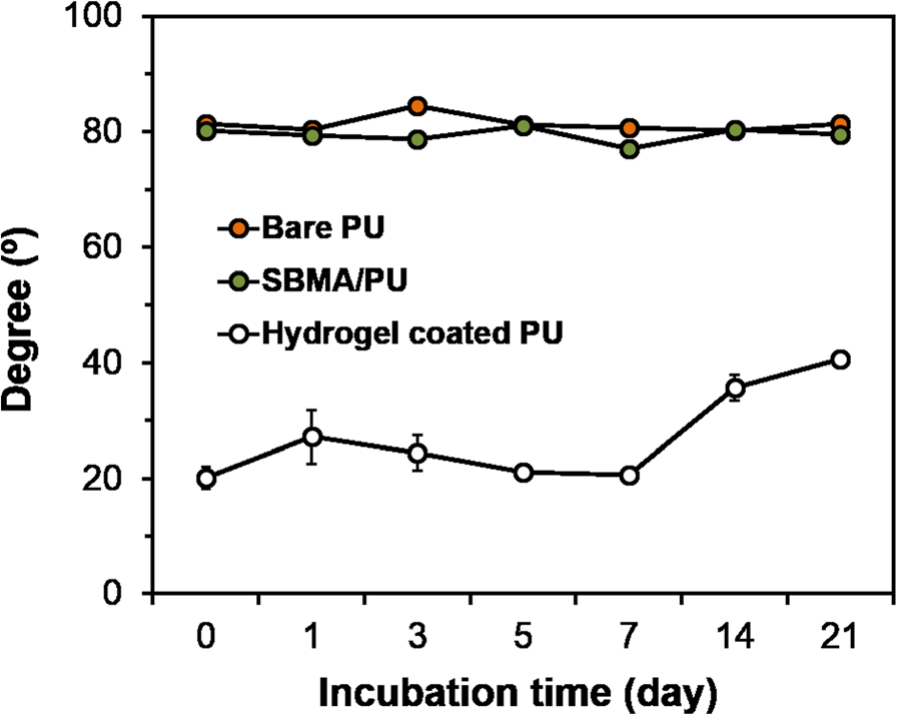
Water contact angle of bare and modified PU surfaces, after incubating in aqueous solution

The stability of the hydrogel coatings was analyzed by measuring the hydrophilicity of modified PU substrates during a 21-day incubation in aqueous solution. It was observed that the SBMA hydrogel-coated PU substrates had a high stability in aqueous condition (Fig. 5), with no marked change in the water contact angle (around 20°) after incubation for 7 days. At this time, the water contact angle of the hydrogel-coated PU surfaces slightly increased up to 40°, but was still lower than that of bare PU surfaces (81°) and PU substrates treated with only SBMA solution (SBMA/PU). From the above results, we can confirm the high stability of the Fenton reaction-mediated coating method.

### In vitro anti-fouling evaluation

#### Fibrinogen adsorption

The protein adsorption of SBMA hydrogel-coated PU substrates was evaluated using HRP-conjugated anti-IgG (by an indirect ELISA). The amount of anti-IgG adsorption on SBMA hydrogel-coated PU substrates was determined by monitoring the increase in tangerine color intensity at 450 nm, caused by the reaction of HRP with TMB, relative to bare PU. The sensitivity of the ELISA method was found to be equivalent to that of the ^125^I–radiolabeled fibrinogen method for estimating an adsorption level [22].

**Fig. 6:**
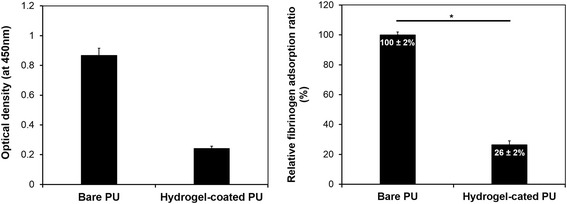
Fibrinogen absorption on bare and hydrogel-coated PU surfaces: optical density of absorbed fibrinogen solution (**a**) and relative fibrinogen absorption ratio, compared to bare surfaces (**b**). *P < 0.0001 vs. bare PU

In this study, the absorbance from the bare PU substrate was set as 100% for calculating the relative adsorption. As shown in Fig. 6, there was a significant decrease in fibrinogen absorption on SBMA hydrogel-coated PU surfaces, compared to the bare PU surfaces. This result confirmed the effect of the zwitterion-based hydrogel with its well-packed structure, to reduce the non-specific protein adsorption on the PU surface. It is well-known that the adsorption of protein on the implant surfaces is the first step in thrombus formation, one of the common and severe events in blood-contacting devices [2]. Therefore, the reduction in fibrinogen absorption, the key protein of the coagulation cascade, making our SBMA hydrogel-coated surface a potential candidate for in vivo biomedical applications.

### Cell adhesion

**Fig. 7:**
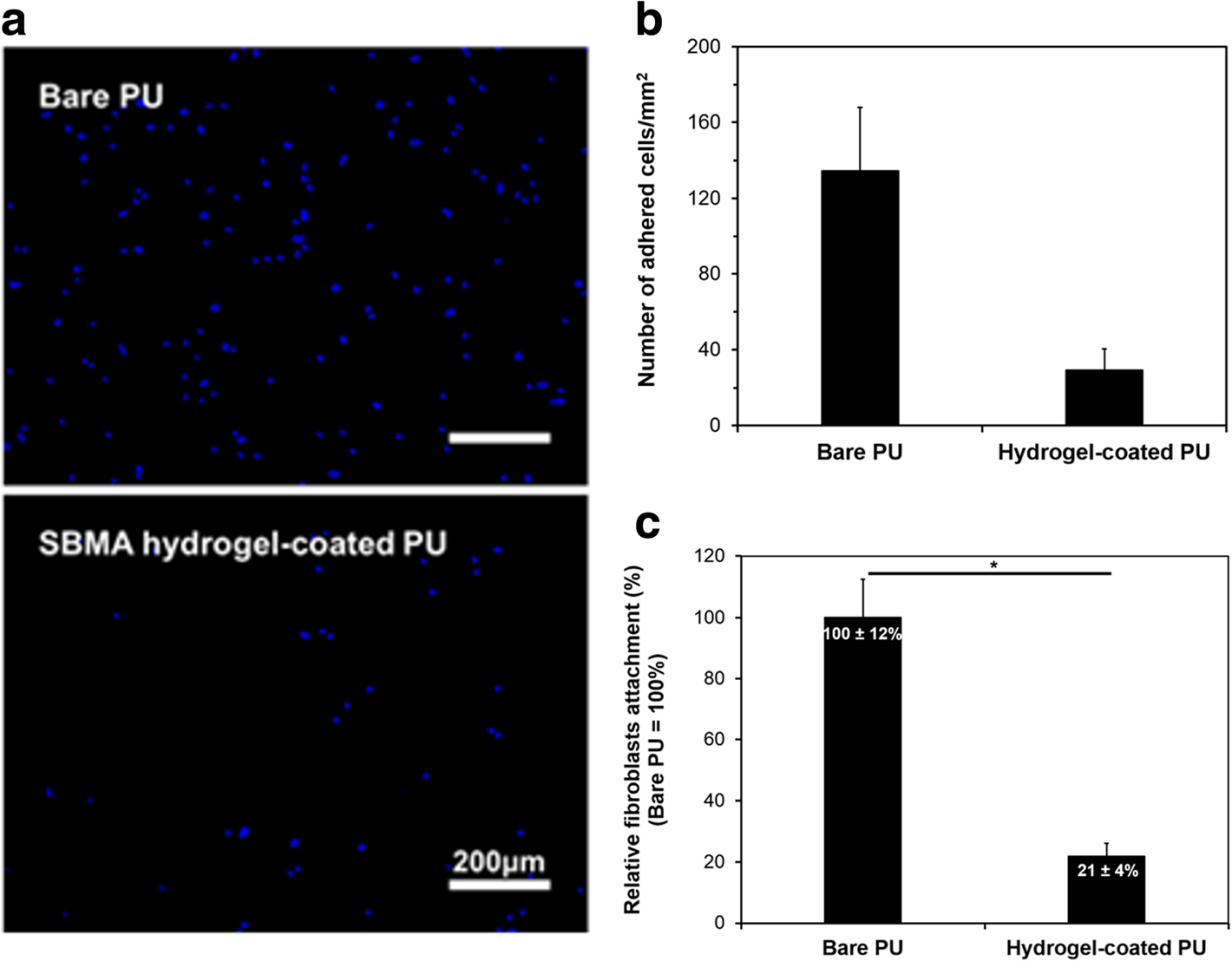
In vitro fibroblast adhesion on bare and hydrogel coated PU surfaces: Fluorescence micrographs of cells adhered to the bare and SBMA hydrogel-coated PU surfaces (**a**); amount of attached cells measured by ImageJ analysis (**b**) and relative cell attachment ratio, compared to bare surfaces (**c**). *P < 0.01 vs. bare PU

The cell attachment response on the material surface plays an important role in designing various types of biomedical devices and usually needs to be prevented by proper surface modification. The cell-repellent properties of the SBMA hydrogel-coated PU surfaces were tested using hDFBs as a model cell line and bare PU as the control. Figure 7a shows the representative fluorescence images of the hDFBs adhered on the bare and SBMA hydrogel-coated PU surfaces. While the cells adhered well to the bare PU surfaces, the SBMA hydrogel-coated PU surfaces showed relatively less adherence. The quantitative statistics of the attached hDFBs was also obtained from ImageJ analysis (Fig. 7b). Compared to the bare PU, the SBMA hydrogel-coated PU surfaces suppressed 80% of adhered hDFBs (Fig. 7c). The reduction in cell adhesion of the hydrogel-coated surfaces may result from the cytotoxicity of the hydrogel coating. However, the excellent biocompatibility of SBMA hydrogels and Fenton cross-linking reaction has been widely demonstrated [6, 19, 23, 24]. In our study, the modified surfaces were washed thoroughly with SDS and immersed in DW overnight, to remove the solvent and unreacted monomers. In addition, the cell-repellent property of our hydrogel-coated surfaces corroborated with and was comparable to previous studies, which reduced over 75% cell attachment relative to bare surfaces [9, 25]. These results are most likely due to the hydration properties and the lack of electrostatic properties of the SBMA hydrogel coating, deeming it resistant to protein absorption before cell attachment.

## Conclusions

In this study, we developed a facile method to polymerize and coat a zwitterionic-based hydrogel onto PU surfaces via the Fenton reaction. By using the two-phase process, the free radicals were formed on the PU surface/reaction solution interface, which improves the efficiency of polymerization. The obtained hydrogel coating showed good adhesion to the PU surface with high stability after 21 days of incubation in aqueous solution. Importantly, the SBMA hydrogel-coated PU surfaces exhibited good resistance against both fibrinogen and fibroblasts, attributed to the excellent anti-fouling properties of zwitterionic molecules. Therefore, the SBMA hydrogel-coated PU surfaces with anti-fouling properties could have great potential for use in biomedical devices.
